# Long noncoding RNA H19 knockdown promotes angiogenesis via IMP2 after ischemic stroke

**DOI:** 10.1111/cns.70000

**Published:** 2024-08-19

**Authors:** Liyuan Zhong, Junfen Fan, Feng Yan, Zhenhong Yang, Yue Hu, Lingzhi Li, Rongliang Wang, Yangmin Zheng, Yumin Luo, Ping Liu

**Affiliations:** ^1^ Institute of Cerebrovascular Disease Research and Department of Neurology Xuanwu Hospital of Capital Medical University Beijing China; ^2^ Beijing Geriatric Medical Research Center and Beijing Key Laboratory of Translational Medicine for Cerebrovascular Diseases Beijing China

**Keywords:** angiogenesis, blood–brain barrier, cerebral ischemia/reperfusion, IMP2, lncRNA H19

## Abstract

**Aims:**

This study aimed to explore the effects of long noncoding RNA (lncRNA) H19 knockdown on angiogenesis and blood–brain barrier (BBB) integrity following cerebral ischemia/reperfusion (I/R) and elucidate their underlying regulatory mechanisms.

**Methods:**

A middle cerebral artery occlusion/reperfusion model was used to induce cerebral I/R injury. The cerebral infarct volume and neurological impairment were assessed using 2,3,5‐triphenyl‐tetrazolium chloride staining and neurobehavioral tests, respectively. Relevant proteins were evaluated using western blotting and immunofluorescence staining. Additionally, a bioinformatics website was used to predict the potential target genes of lncRNA H19. Finally, a rescue experiment was conducted to confirm the potential mechanism.

**Results:**

Silencing of H19 significantly decreased the cerebral infarct volume, enhanced the recovery of neurological function, mitigated BBB damage, and stimulated endothelial cell proliferation following ischemic stroke. Insulin‐like growth factor 2 mRNA‐binding protein 2 (IMP2) is predicted to be a potential target gene for lncRNA H19. H19 knockdown increased IMP2 protein expression and IMP2 inhibition reversed the protective effects of H19 inhibition.

**Conclusion:**

Downregulation of H19 enhances angiogenesis and mitigates BBB damage by regulating IMP2, thereby alleviating cerebral I/R injury.

## Graphical abstract

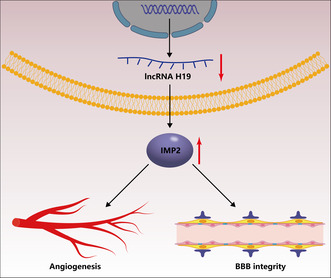

## 1 INTRODUCTION

Ischemic stroke (IS), which refers to the necrosis and softening of brain tissue caused by cerebral ischemia and hypoxia due to the interruption of blood supply to the brain, is among the main causes of long‐term neurological deficits and death among middle‐aged and older adults globally.^1^, ^2^ Clinically, the main objective of treating ischemic stroke is to restore blood supply. Currently, mechanical thrombectomy, recognized as a principal method for restoring cerebral blood flow, has become the standard treatment for patients experiencing large vessel occlusions. Reperfusion is useful for restoring blood supply, but it inevitably induces additional tissue damage. According to previous reports, cerebroprotective agents in the prehospital environment are crucial to limiting the enlargement of infarcts until the patient can undergo reperfusion therapy.^3^ Therefore, there is an urgent need to identify cerebroprotective agents against IS.

Long noncoding RNAs (lncRNAs) are a subtype of noncoding RNAs with more than 200 nucleotides and have no protein encoding ability.^4^ Extensive research on lncRNAs has revealed their impact on the occurrence, progression, and prognosis of IS. A previous study using microarray chips identified a substantial number of abnormally expressed lncRNAs in the rat cortex after cerebral ischemic injury.^5^ Abnormal lncRNA expression was detected in the peripheral blood of patients who suffered IS.^6^ This indicates that some lncRNAs may serve as promising novel therapeutic targets for IS. As one of the first imprinted genes discovered, lncRNA H19 demonstrates a high level of conservation throughout evolution. Researchers have assessed the expression patterns and stability of lncRNAs in the cortex, white matter, and cerebellum using brain tissue from five autopsies.^7^ Their findings revealed that H19 ranks among the top 10 lncRNAs with stable expression in the human brain.^7^ The involvement of lncRNA H19 in atherosclerosis, through its targeting of ACP5, has been linked to an elevated risk of IS.^8^ A clinical investigation demonstrated that individuals carrying the H19 rs217727 gene polymorphism had a higher susceptibility to IS, suggesting that circulating lncRNA H19 can be regarded as a novel biomarker and a prospective therapeutic target for individuals with IS.^9^ In a prior experimental investigation, lncRNA H19 expression in the circulating blood of patients with IS was elevated. This elevation of expression was positively related to stroke severity, as measured by the National Institutes of Health Stroke Scale scores on days 7, 30, and 90 after IS. This indicated a close association between the level of lncRNA H19 expression and the prognosis of IS.^10^ A separate study also revealed that patients with IS who exhibited increased plasma concentrations of H19 had a heightened risk of aspirin resistance, potentially leading to recurrent ischemic strokes.^11^ These previous studies have established the vital role of lncRNA H19 in IS and have suggested that H19 inhibitors are viable cerebroprotective agents for IS.

A previous study reported that improved angiogenesis around damaged areas in the brain after stroke was linked to a longer duration of survival and improved brain function.^12^ The formation of the blood–brain barrier (BBB) begins with blood vessel formation, and its integrity is crucial for maintaining the internal balance of the brain and protecting the nervous system.^13^ BBB disruption plays a pivotal role in the advancement of pathophysiological processes and significantly affects the prognosis of ischemic stroke.^14^ Hence, angiogenesis and BBB restoration can promote brain repair and lead to long‐lasting functional recovery following ischemic stroke.

However, the impact of lncRNA H19 on angiogenesis and the BBB following cerebral ischemia and its underlying regulatory pathways remains unknown despite extensive investigations over the years. This study aimed to investigate the efficacy of lncRNA H19 downregulation for cerebroprotection in IS and its potential regulatory mechanisms to offer novel insights into IS treatment.

## 2 MATERIALS AND METHODS

### 2.1 Animals

The animal experimental protocols were approved by the Animal Experiments Ethics Committee of Xuanwu Hospital, Capital Medical University. Male C57BL/6 J mice weighing 21–23 g (8–9 weeks old) were obtained from Vital River Laboratory Animal Technology Co., Ltd. and were cared for according to the guidelines outlined in the National Institutes of Health Guide for the Care and Use of Laboratory Animals. Throughout the experiment, all the mice were provided ad libitum access to food and water. A 12 h light and 12 h dark cycle was maintained, and the lights were turned on at 7:00 AM.

### 2.2 Intracerebroventricular injection

For H19 overexpression and knockdown, mouse H19‐overexpression adenovirus or H19 short hairpin (sh) RNA (shRNA) lentivirus was injected intracerebroventricularly. Mouse adenoviruses and lentiviruses were purchased from Hanheng Biological Company. The sequences of sh‐H19, sh‐IMP2, and sh‐NC were as follows: 5’‐CCCUCAAGAUGAAAGAAAUTT‐3’ (sh‐H19), 5’‐GGGUAAAGUGGAAUUGCAUTT‐3’ (sh‐IMP2), 5’‐UUCUCCGAACGUGUCACGUTT‐3’ (sh‐NC). The mice described above, except for the sham group, were anesthetized with approximately 4.0%–5.0% enflurane and maintained with 1.5%–3.5% enflurane in a mixture of 70% N_2_O and 30% O_2_. After anesthetization, the mice were fixed in the prone position using a brain stereotaxic frame to reduce head movement during the intracerebroventricular injection. A scalp incision was made along the midline of the head skin to expose the skull, followed by drilling a hole in the skull at stereotaxic coordinates 1 mm posterior and 1 mm lateral to the bregma with a depth of 2.5 mm. The adenovirus or lentivirus was administered into the lateral ventricle, with the needle left in place for 5 min to prevent liquid leakage. The needle was withdrawn cautiously and slowly, and the wound was sealed with bone wax. Finally, the skin incisions were individually sutured using interrupted nylon sutures.

### 2.3 Middle cerebral artery occlusion/reperfusion mouse model

Middle cerebral artery occlusion (MCAO)/reperfusion (MCAO/R) surgery was performed to induce cerebral ischemia/reperfusion (I/R) injury using the intraluminal filament method as detailed in a previous study.^15^ C57BL/6J mice were anesthetized with 4.0%–5.0% enflurane. The anesthesia was maintained with 1.5%–3.5% enflurane in a mixture of 70% N_2_O and 30% O_2_. The mice were positioned supine on a heated platform with their limbs secured to the table after the anesthesia. Fur was shaved from the surgical site using an electric razor to expose the neck skin, which was disinfected with povidone‐iodine and 70% ethanol before surgery. The mice were administered lidocaine (3.5 mg/kg, Tiancheng Pharmaceutical Co., Ltd.) at the incision site before the procedure as preemptive analgesia. They received a subcutaneous injection of buprenorphine (0.05 mg/kg, Pharmaceutical Research Institute Pharmaceutical Co., Ltd.) after the surgery, and another dose was administered the following day. A 1.0–1.5‐cm‐long dorsal midline incision was made through the skin on the neck, and the connective tissue was gently separated from the muscle by blunt dissection with Dumont forceps. The right common carotid artery (CCA), internal carotid artery (ICA), and external carotid artery were exposed and carefully separated under a microscope. The superior thyroid and the occipital arteries were electrocoagulated. The ICA and CCA were temporarily closed using microvascular clips, and the far end of the external carotid artery was ligated using surgical nylon monofilaments. A small “V” incision was made using ophthalmic scissors approximately 4 mm away from the bifurcation of the CCA. Next, a monofilament nylon suture coated with silicone rubber (Catalogue number: 701956PK5Re; Doccol Corporation) was gently pushed from the CCA “V” incision to the ICA to occlude the middle cerebral artery (MCA). The nylon suture was gently pushed until a mild resistance was felt (9–10 mm from MCA bifurcation). The wound was closed after confirming hemostasis using nylon sutures. Cerebral blood flow (CBF) was restored by removing the nylon suture to simulate cerebral ischemia/reperfusion injury in vivo after 45 min of MCAO. In the sham group, the common carotid artery was exposed and isolated without ligation or hypoxia. A heating pad was used to maintain the rectal temperature of the mice at approximately 37.0 ± 0.5°C throughout the entire procedure.

### 2.4 Drug administration

Bromo‐2′‐deoxyuridine (BrdU, B‐5002, Sigma) was prepared in sterile physiological saline. Mice were administered BrdU (50 mg/kg) intraperitoneally once daily for seven consecutive days post‐cerebral I/R injury.

### 2.5 Laser speckle imaging

After anesthetization, the mice were placed in the prone position with their head fixed in a brain stereotaxic frame to minimize brain motion during the imaging procedure. The hair above the scalp was shaved and disinfected with povidone‐iodine and 70% ethanol, and a cut was made in the scalp along the midline of the head to reveal the skull, which was cleaned and moistened carefully with sterile saline. Subsequently, the mice were placed in the prone position under transcranial laser Doppler (LDF, PeriFlux System 5000; Perimed, Sweden) to observe the CBF images. The position of the mice and the relative humidity of the skull surface were adjusted according to the CBF images. Global and regional CBF were monitored and recorded when upon reaching a relatively steady state. Two regions of interest (ROIs) were delineated symmetrically within the MCA, resulting in the acquisition of the left and right ROIs. Subsequently, the relative CBF (rCBF) was computed using the following formula:

$$\text{rCBF}=\frac{\mathrm{CBF}\;\text{value for}\;\mathrm{ROI}2}{\mathrm{CBF}\;\text{value for}\;\mathrm{ROI}1}$$

where *rCBF* is the relative CBF, *ROI1* is the left ROI, and *ROI2* is the right ROI.

### 2.6 Balance beam walking test

The mice underwent a balance beam walking test to evaluate their exercise capacity, focusing on motor coordination and integration functions, following previously described protocols.^16^ A wooden beam measuring 120 cm long and 2 cm wide was placed 30 cm above the bench surface. The mice were gently removed from their home cages and placed at the starting point of the balance beam. Each mouse was trained to cross the beam three times per day for five consecutive days before MCAO treatment. The number of feet (both hind and front legs) moving completely from the top of the beam was recorded and assessed. The mice were given sufficient time (approximately 5 min) to cross the beams. The experiment was performed three times, and the outcomes were averaged. The mice were allowed to rest for 2–3 min between sessions. The beam was cleaned with 70% ethanol and water to prevent olfactory cues.

Motor performance was rated using the following scale: 0 = mice were unable to remain on the balance beam; 1 = mice remained stationary on the balance beam; 2 = mice attempted to traverse the balance beam but were unsuccessful; 3 = mice faced challenges crossing the balance beam with over 50% foot slips; 4 = mice crossed the balance beam with more than 1‐foot slip but less than 50%; 5 = mice successfully crossed the balance beam with only 1‐foot slip; and 6 = mice successfully crossed the balance beam without any foot slips.

### 2.7 Modified neurological severity score

To evaluate neurological recovery after MCAO treatment, the modified neurological severity score (mNSS) was performed as previously described.^17^ The mNSS is a classic and common behavioral test for comprehensively assessing neurological function; it includes tail suspension, walk tests, balance beam tests, sensory tests, reflex absence, and abnormal movements. The mNSS score ranges from 0 to 18 points, with 0 representing a normal status, 1–6 points indicating mild injury, 7–12 points signifying moderate injury, and 13–18 points indicating severe injury.

### 2.8 Staining with 2,3,5‐triphenyl‐tetrazolium chloride

The mice were anesthetized with amobarbital (10%, 0.5 mL/100 g) and transcardially perfused with 0.9% saline on day 7 post‐MCAO surgery. Following perfusion, the mice were decapitated and their brains were sectioned into 1‐mm‐thick coronal slices. These brain slices were immersed in a 2% 2,3,5‐triphenyl‐tetrazolium chloride (TTC) solution (Sigma‐Aldrich) for 10 min at 37°C in a dark incubator, followed by fixation in 4% paraformaldehyde for infarct volume assessment. The viable brain tissue appeared red after TTC staining, whereas the infarcted tissue appeared white. The infarct volume was quantified using Image J software, and the percentage of the ischemic lesion area was calculated using the formula:

$$\text{Infarct volume}\;\left(\%\right)=\frac{\text{contralateral hemisphere volume}–\text{non}-\text{infarct ipsilateral hemisphere volume}}{\text{contralateral hemisphere volume}\;}x100\%$$

### 2.9 Western blotting

Proteins were separated from the cortex of the infarcted hemisphere for western blot analysis. Next, 20 μg protein samples were resolved using 10% sodium dodecyl sulfate‐polyacrylamide gel electrophoresis and transferred onto a polyvinylidene fluoride (PVDF) membrane. The PVDF membrane was blocked with bovine serum albumin for 2 h at room temperature, followed by overnight incubation with the following appropriately diluted primary antibodies at 4°C: anti‐CD31 (1:500, Servicebio), anti‐VEGFR2 (1:1000, CST), anti‐IMP2 (1:500, Proteintech), anti‐claudin5 (1:500, Bioss), anti‐ZO‐1 (1:500, Proteintech), and anti‐β‐actin (1:1000, Santa Cruz). The PVDF membranes were incubated with horseradish peroxidase‐conjugated secondary antibodies (1:2000; Santa Cruz) at room temperature for 1 h. Finally, the immunoblots were visualized using an enhanced luminescence kit (Millipore), and the integrated density values were determined using ImageJ software (National Institutes of Health). The relative level of protein expression was determined by measuring the optical density in comparison to the control group, with the values being normalized against the optical density of β‐actin.

### 2.10 Immunofluorescence staining

The mice were anesthetized using amobarbital (10%, 0.5 mL/100 g) and transcardially perfused with 0.9% saline. They were decapitated and their brains were fixed in 10% neutral buffered formalin (Fisher Scientific). The fixed brains were sliced coronally and prepared for paraffin embedding. The brain tissue sections were treated with 3% donkey serum for 1 h at room temperature for blocking. This was followed by immunostaining with the following specific primary antibodies in a humidified chamber for 12 h at 4°C: anti‐VEGFR2 (1:200, Proteintech), anti‐CD31 (1:200, Santa Cruz), anti‐BrdU (1:200, Proteintech), and anti‐IMP2 (1:200, Proteintech). They were subsequently incubated with fluorescent‐conjugated secondary antibodies (1:200, Santa Cruz).

### 2.11 Statistical analysis

The statistical analyses were conducted using the computer software SPSS, version 27.0 (SPSS Inc.) and GraphPad Prism 9.0 (GraphPad Software). The Shapiro–Wilk (SW) test was used to evaluate the normality of the data distributions. The normally distributed data were analyzed using Student's *t*‐test for two comparisons or one‐way ANOVA with Tukey's post hoc test for multiple comparisons. For non‐normally distributed data, the Mann–Whitney *U* test was employed as an alternative to the *t*‐test, and the Kruskal–Wallis test was selected as a replacement for ANOVA. The results were considered statistically significant when their *p*‐values were less than or equal to 0.05.

## 3 RESULTS

### 3.1 Upregulation of H19 increases the cerebral infarct volume and aggravates neurological damage after cerebral I/R injury

**FIGURE 1:**
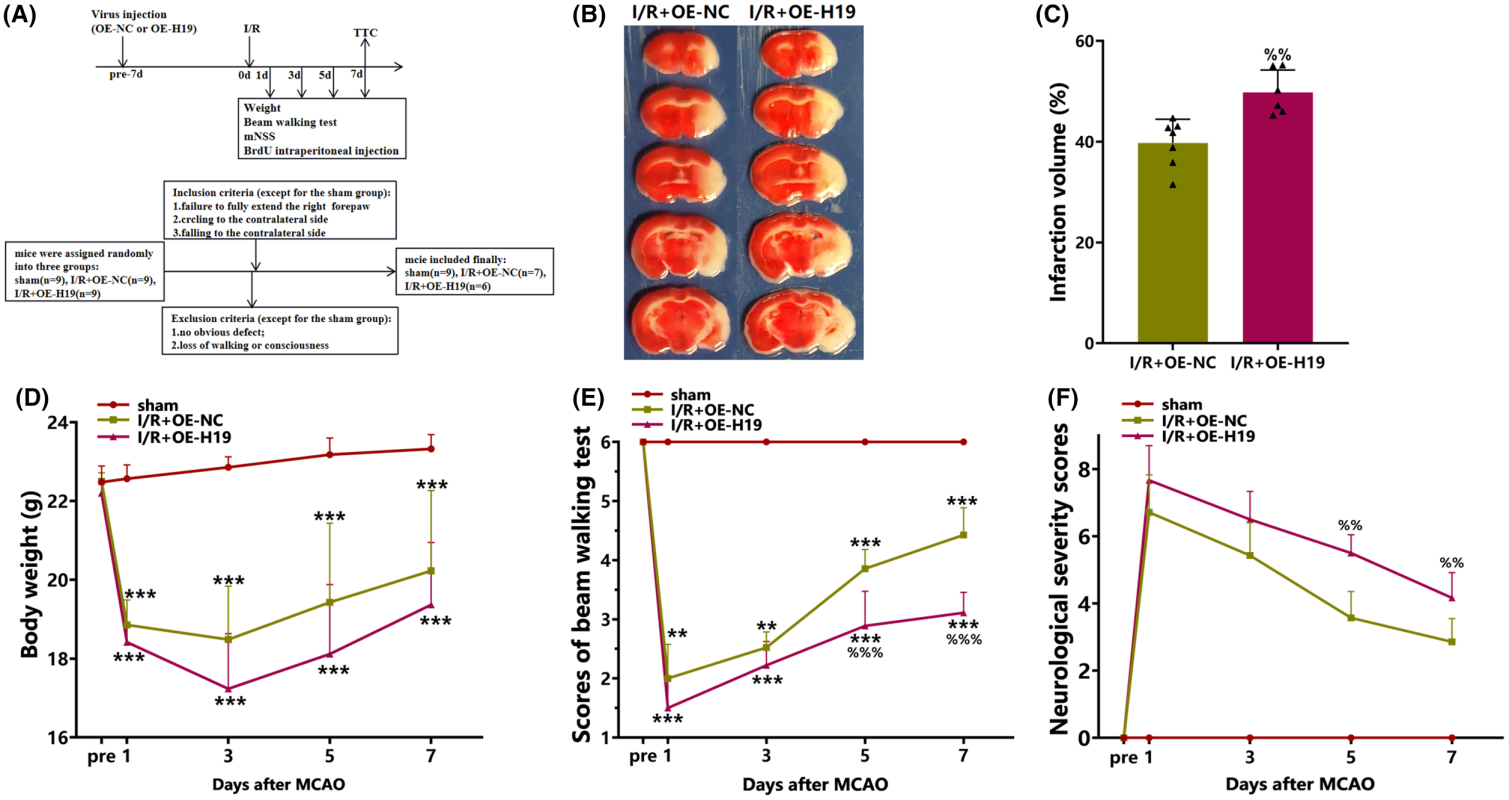
H19 overexpression increased the cerebral infarct volume and aggravated neurological damage. (A) Experimental design and inclusion/exclusion criteria; (B) Representative TTC staining images; (C) Quantification of TTC staining (data were analyzed using t test; *N* = 7/I/R + OE‐NC group, *N* = 6/I/R + OE‐H19 group); (D) Weight changes of mice in each group (data were analyzed using ANOVA with Tukey post hoc test; *N* = 9/sham group, *N* = 7/I/R + OE‐NC group, *N* = 6/I/R + OE‐H19 group); (E) Beam walking test (day 1 and day 3: Data were analyzed using Kruskal–Wallis test; day 5 and day 7: Data were analyzed using ANOVA with Tukey post hoc test; *N* = 9/sham group, *N* = 7/I/R + OE‐NC group, *N* = 6/I/R + OE‐H19 group); (F) Modified neurological severity score (mNSS, day 1 and day 7: Data were analyzed using t test; day 3 and day 5: Data were analyzed using Mann–Whitney *U* test; *N* = 9/sham group, *N* = 7/I/R + OE‐NC group, *N* = 6/I/R + OE‐H19 group). Data are presented as the mean ± S.D. ** < 0.01 and ***p < 0.001 vs. sham group. %%*p* < 0.01 and %%%p < 0.001 vs. I/R + OE‐NC group.

The TTC staining results demonstrated a significant increase in the percentage of cerebral infarct volume due to H19 overexpression relative to that of the I/R + OE‐NC group on day 7 after cerebral I/R (*p* < 0.01; Figure 1B,C).

The mice in the I/R + OE‐H19 group showed poorer neurological functional recovery post‐I/R injury than those in the I/R + OE‐NC group, as evidenced by increased foot slips (both hind and front legs) during the beam balance test on days 5 (*p* < 0.001, Figure 1E) and 7 (*p* < 0.001, Figure 1E) after cerebral I/R injury. Furthermore, the mNSS scores of the mice in the I/R + OE‐H19 group were significantly higher than those of the mice in the I/R + OE‐NC group on days 5 (*p* < 0.01, Figure 1F) and 7 (*p* < 0.01, Figure 1F) after cerebral I/R injury.

### 3.2 Knockdown of H19 reduces the volume of cerebral infarction and mitigates neurological impairment after cerebral I/R injury

**FIGURE 2:**
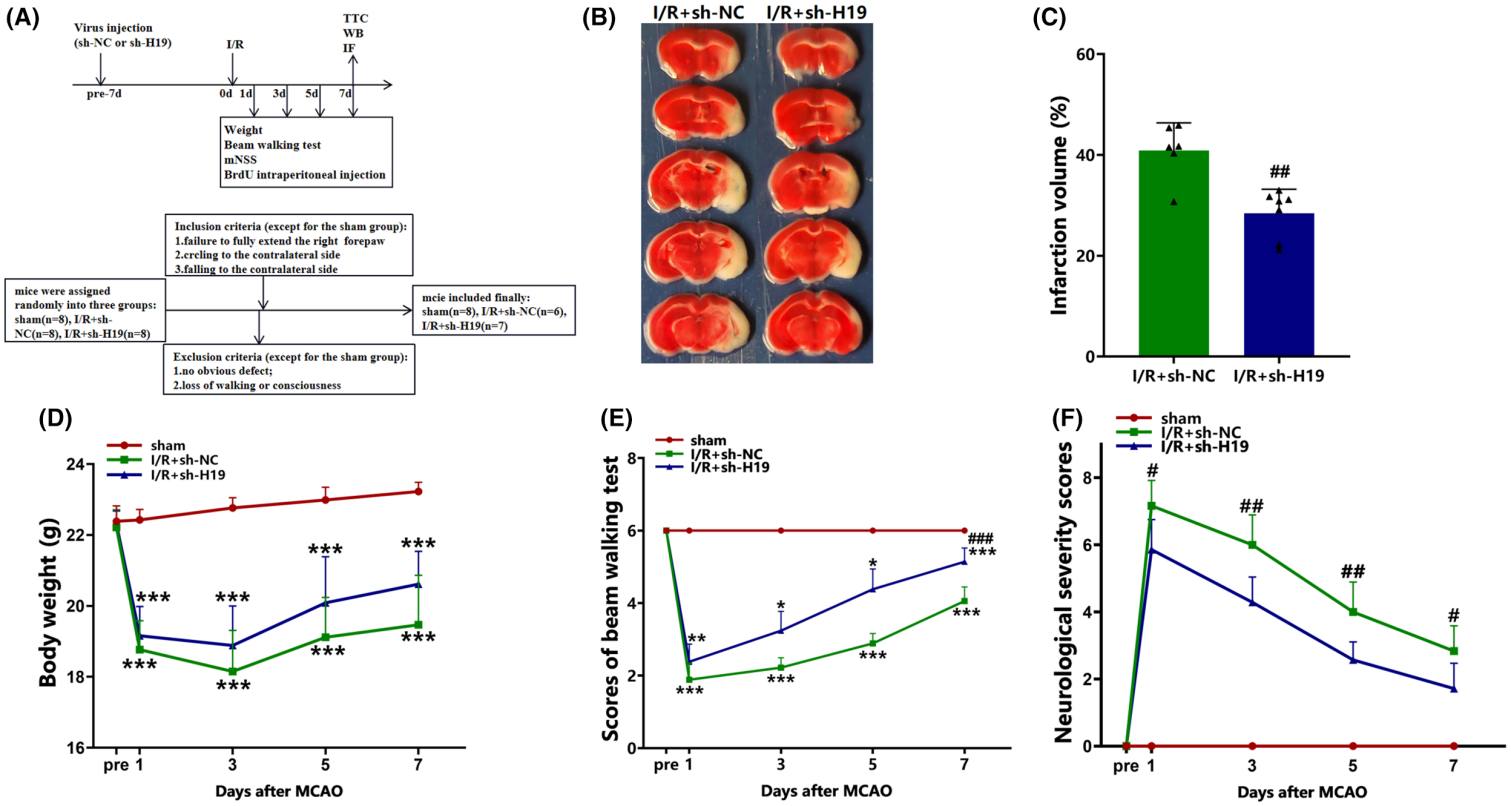
Knockdown of lncRNA H19 reduced the volume of cerebral infarction and promoted the recovery of neurological function after I/R. (A) Experimental design and inclusion/exclusion criteria; (B) Representative TTC staining images; (C) Quantification of TTC staining (data were analyzed using t test; *N* = 6/I/R + sh‐NC group, *N* = 7/I/R + sh‐H19 group); (D)Weight changes of mice in each group (data were analyzed using ANOVA with Tukey post hoc test; *N* = 8/sham group, *N* = 6/I/R + sh‐NC group, *N* = 7/I/R + sh‐H19 group); (E) Beam walking test (day 1, day 3, and day 5: Data were analyzed using Kruskal–Wallis test; day 7: Data were analyzed using ANOVA with Tukey post hoc test; *N* = 8/sham group, *N* = 6/I/R + sh‐NC group, *N* = 7/I/R + sh‐H19 group); (F) Modified neurological severity score (mNSS, day 1, day 3, and day 7: Data were analyzed using *t* test; day 5: Data were analyzed using Mann–Whitney *U* test; *N* = 8/sham group, *N* = 6/I/R + sh‐NC group, *N* = 7/I/R + sh‐H19 group). Data are presented as the mean ± S.D. *p < 0.05, ** < 0.01, and ***p < 0.001 vs. sham group. #*p* < 0.05, ##*p* < 0.01, and ##*p* < 0.001 vs. I/R + sh‐NC group.

TTC staining demonstrated a significant decrease in brain infarction volume due to H19 knockdown relative to the observations in the I/R + sh‐NC group on day 7 post‐cerebral I/R (*p* < 0.01; Figure 2B,C).

Mice in the I/R + sh‐H19 group showed enhanced neurological functional recovery following cerebral ischemia relative to those in the I/R + sh‐NC group, as indicated by reduced foot slips (both hind and front legs) during the beam balance test on day 7 after cerebral ischemic injury (*p* < 0.001; Figure 2E). Additionally, mice treated with sh‐H19 exhibited significantly lower mNSS scores than those treated with sh‐NC on days 1 (*p* < 0.05, Figure 2F), 3 (*p* < 0.01, Figure 2F), 5 (*p* < 0.01, Figure 2F), and 7 (*p* < 0.05, Figure 2F) post‐I/R injury.

### 3.3 Knockdown of H19 increases the cerebral blood flow after cerebral I/R injury

**FIGURE 3:**
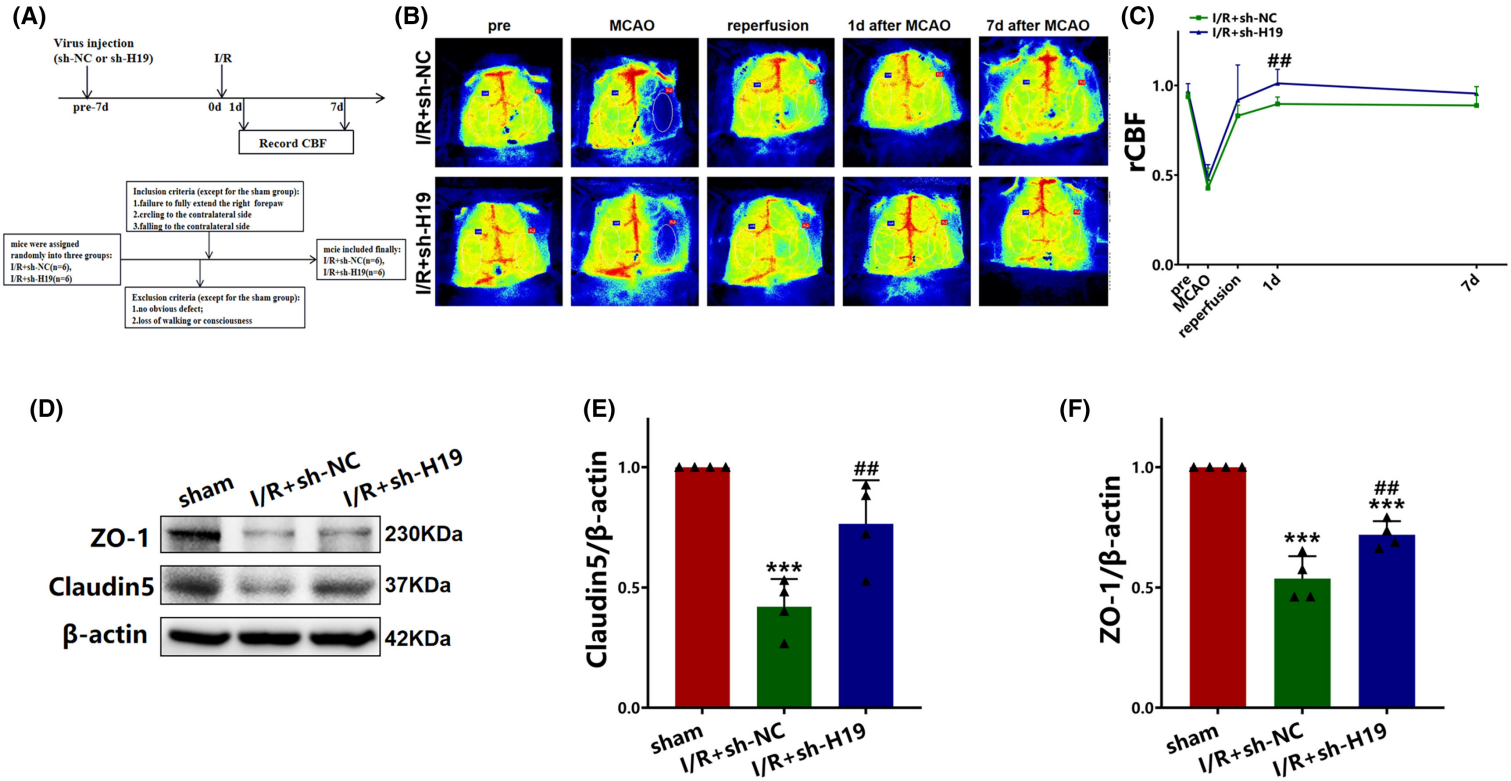
Evaluation of the effect of H19 inhibition on CBF and BBB integrity after cerebral I/R. (A) Experimental design and inclusion/exclusion criteria; (B)Representative images of CBF changes in each group; (C) The relationship between rCBF change and time (pre, MCAO, 1d and 7d: Data were analyzed using t‐test; reperfusion: Data were analyzed using Mann–Whitney *U* test; *N* = 6); (D) Expression of ZO‐1 and claudin5 detected by western blot; (E) Quantification analysis of claudin5 analyzed by western blot (data were analyzed using ANOVA with Tukey post hoc test; *N* = 4); (F) Quantification analysis of ZO‐1 analyzed by western blot (data were analyzed using ANOVA with Tukey post hoc test; *N* = 4). Data are presented as the mean ± S.D. ***p < 0.001 vs. sham group. ##*p* < 0.01 vs. I/R + sh‐NC group.

Representative and quantified images of rCBF are shown in Figure 3B,C. No significant color difference in infarct size was observed between the I/R + sh‐NC and I/R + sh‐H19 groups before MCAO, indicating a similar cerebral blood flow status (Figure 3B). The proportions of the red area on the surgical side were lower than those on the contralateral normal side during the ischemic period for both groups (Figure 3B). Following reperfusion, the proportion of the red area on the surgical side increased significantly but remained lower than that on the contralateral normal side (Figure 3B). Subsequently, the proportion of the red area on the surgical side increased on days 1 and 7 post‐reperfusion from that at the reperfusion time point (Figure 3B).

The rCBF‐time curve in Figure 2C provides details on the rCBF differences between the ROIs of the two groups. The sh‐H19 group exhibited a significant increase in rCBF relative to the I/R + sh‐NC group on day 1 post‐reperfusion (*p* < 0.01; Figure 3C). However, there were minimal differences between the rCBF for the ROIs of the I/R + sh‐NC and I/R + sh‐H19 groups on day 7 post‐reperfusion (*p* > 0.05; Figure 3C).

### 3.4 Knockdown of H19 alleviates BBB disruption after cerebral I/R injury

Western blot analysis demonstrated a significant decrease in the claudin5 (*p* < 0.001; Figure 3D,E). and ZO‐1 (*p* < 0.001; Figure 3D,F) concentrations of the I/R + sh‐NC group relative to those of the sham group. Administration of sh‐H19 led to an increase in the levels of expression of claudin5 (*p* < 0.01; Figure 3D,E) and ZO‐1 (*p* < 0.01; Figure 3D,F) relative to those for the I/R + sh‐NC group. These suggest that inhibiting H19 resulted in elevated concentrations of tight junction proteins, such as claudin5 and ZO‐1, after I/R injury, indicating the mitigation of BBB disruption after H19 knockdown.

### 3.5 Knockdown of H19 promotes angiogenesis after cerebral I/R injury

**FIGURE 4:**
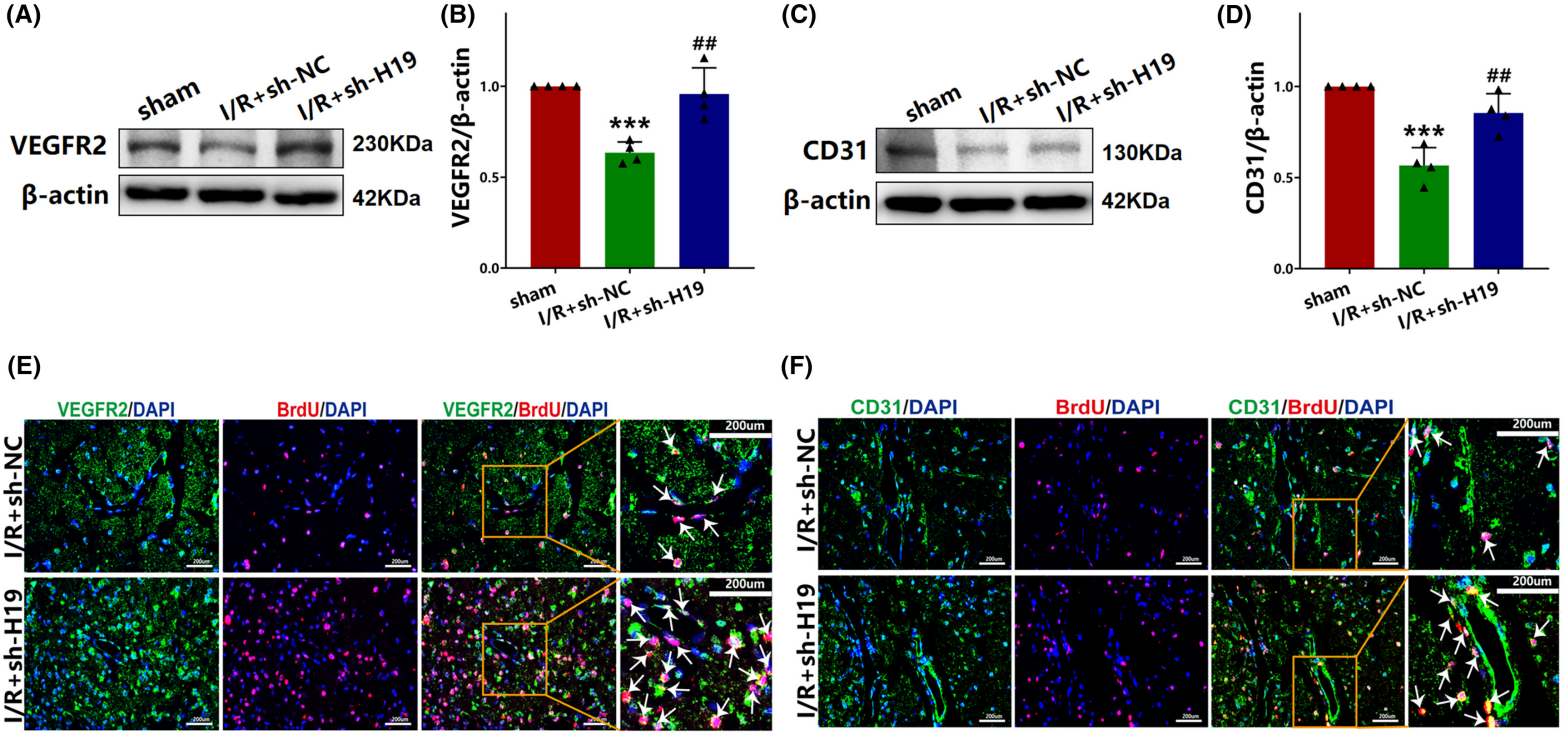
Knockdown of lncRNA H19 promoted angiogenesis after cerebral ischemia/reperfusion. (A) Expression of VEGFR2 detected by western blot; (B) Quantification analysis of VEGFR2 analyzed by western blot (data were analyzed using ANOVA with Tukey post hoc test; *N* = 4); (C) Expression of CD31 detected by western blot; (D) Quantification analysis of VEGFR2 analyzed by western blot (data were analyzed using ANOVA with Tukey post hoc test; *N* = 4); (E) Double immunofluorescence staining of BrdU (red) and VEGFR2 (green); (F) Double immunofluorescence staining of BrdU (red) and CD31 (green). Data are presented as the mean ± S.D. ***p < 0.001 vs. sham group. ##*p* < 0.01 vs. I/R + sh‐NC group.

Western blot analysis revealed that the concentration of VEGFR2 (*p* < 0.001; Figure 4A,B) and CD31 (*p* < 0.001; Figure 4C,D) were significantly decreased in the I/R + sh‐NC group than in the sham group. In contrast, the concentration of VEGFR2 (*p* < 0.01; Figure 4A,B) and CD31 (*p* < 0.01; Figure 4C,D) were increased in the I/R + sh‐H19 group relative to that in the I/R + sh‐NC group. Immunofluorescent staining indicated a higher number of BrdU+/VEGFR2+ (Figure 4E) and BrdU+/CD31+ (Figure 4F) cells in the I/R + sh‐H19 group than in the I/R + sh‐NC group.

### 3.6 Knockdown of H19 increases IMP2 protein expression after cerebral I/R injury

**FIGURE 5:**
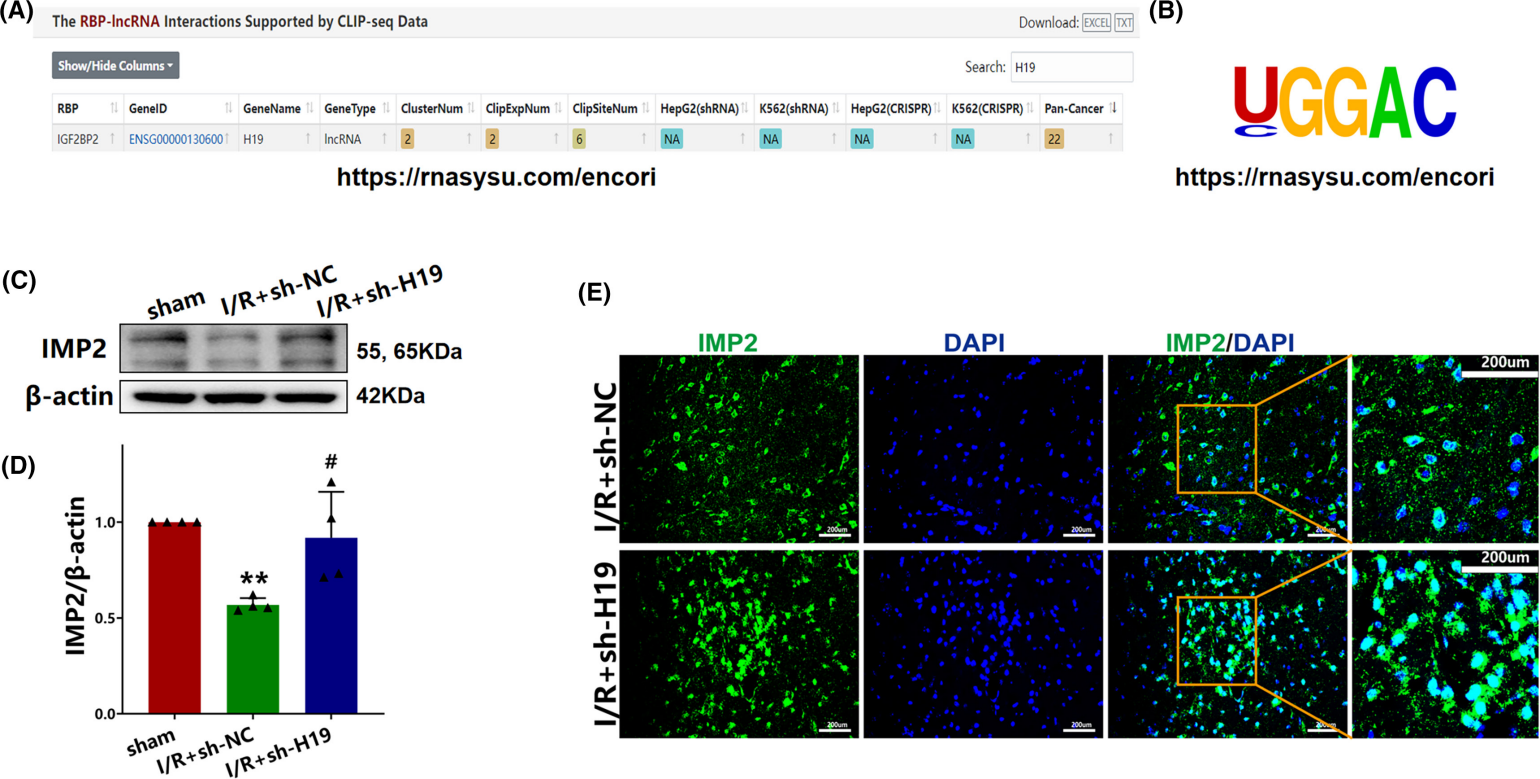
The relationship between lncRNA H19 and IMP2. (A) Starbase predicted that lncRNA H19 could target IMP2; (B) The potential IMP2 target motif in lncRNA H19; (C) Expression of IMP2 detected by western blot; (D) Quantification analysis of IMP2 analyzed by western blot (data were analyzed using ANOVA with Tukey post hoc test; *N* = 4); (E) Immunofluorescence staining of IMP2 after cerebral I/R injury. **p < 0.01 vs. sham group. #*p* < 0.05 vs. I/R + sh‐NC group.

Using an online prediction website ENCORI (https://rnasysu.com/encori), IMP2 was identified as a potential target gene of lncRNA H19 (Figure 5A,B). Western blot analysis revealed a significant increase in IMP2 concentrations in the I/R + sh‐H19 group relative to that in the I/R + sh‐NC group (*p* < 0.05; Figure 5C,D). Immunofluorescence results further supported these findings, showing a higher number of IMP2‐positive cells in the I/R + sh‐H19 group than in the I/R + sh‐NC group (Figure 5E). Collectively, these results indicate a regulatory relationship between lncRNA H19 and IMP2.

### 3.7 Knocking down IMP2 offset the protective effectiveness of H19 silencing after cerebral I/R injury

**FIGURE 6:**
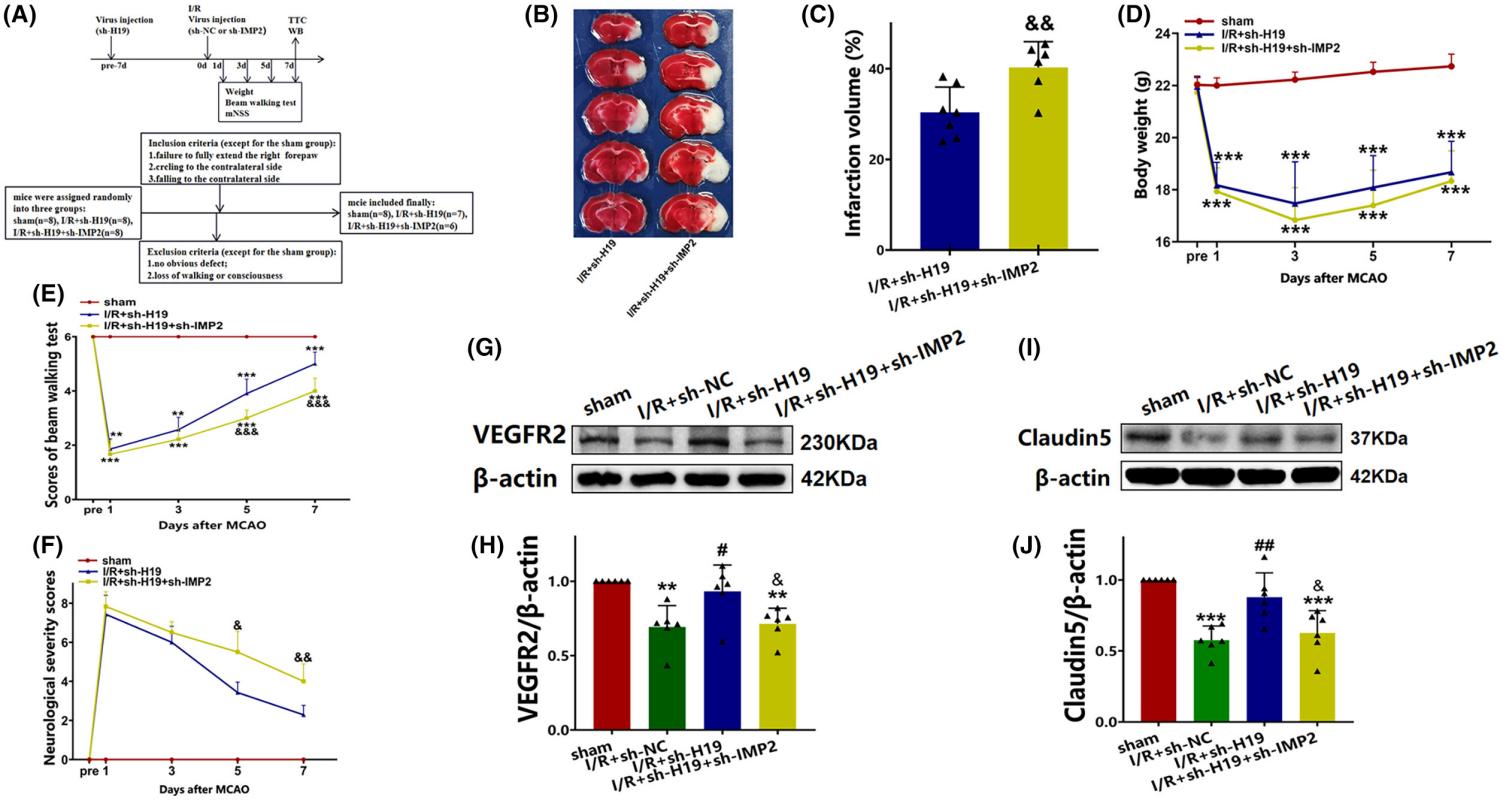
IMP2 inhibition reversed the effect of H19 knockdown on mice following cerebral ischemic injury. (A) Experimental design and inclusion/exclusion criteria; (B) Representative TTC staining images; (C) Quantification of TTC staining (Data were analyzed using *t* test; *N* = 7/I/R + sh‐H19 group, *N* = 6/I/R + sh‐H19 + sh‐IMP2 group); (D) Weight changes of mice in each group (data were analyzed using ANOVA with Tukey post hoc test; *N* = 8/sham group, *N* = 7/I/R + sh‐H19 group, *N* = 6/I/R + sh‐H19 + sh‐IMP2 group); (E) Beam walking test (day 1 day 3: Data were analyzed using Kruskal–Wallis test; day 5 and day 7: Data were analyzed using ANOVA with Tukey post hoc test; *N* = 8/sham group, *N* = 7/I/R + sh‐H19 group, *N* = 6/I/R + sh‐H19 + sh‐IMP2 group; (F) Modified neurological severity score (mNSS, day 1: Data were analyzed using *t* test; day 3, day 5, and day 7: Data were analyzed using Mann–Whitney *U* test; *N* = 8/sham group, *N* = 7/I/R + sh‐H19 group, *N* = 6/I/R + sh‐H19 + sh‐IMP2 group); (G) Expression of VEGFR2 detected by western blot; (H) Quantification analysis of VEGFR2 analyzed by western blot (data were analyzed using ANOVA with Tukey post hoc test; *N* = 6); (I) Expression of claudin5 detected by western blot; (J) Quantification analysis of claudin5 analyzed by western blot (data were analyzed using ANOVA with Tukey post hoc test; *N* = 6). Data are presented as the mean ± S.D. **p < 0.01 and ***p < 0.001 vs. sham group. #*p* < 0.05 and ##*p* < 0.01 vs. I/R + sh‐NC group. &*p* < 0.05, &&*p* < 0.01, and &&&*p* < 0.001 vs. I/R + sh‐H19 group.

TTC staining revealed a significantly larger volume of cerebral infarction in the I/R + sh‐H19 + sh‐IMP2 group than in the I/R + sh‐H19 group (*p* < 0.01; Figure 6B,C). Mice in the I/R + sh‐H19 + sh‐IMP2 group exhibited more frequent forelimb or hind limb foot‐slips than I/R + sh‐H19 mice on days 5 (*p* < 0.001; Figure 6E) and 7 (*p* < 0.001; Figure 6E) after ischemic injury. Additionally, mice in the I/R + sh‐H19 + sh‐IMP2 group had higher mNSS scores on days 5 (*p* < 0.05; Figure 6F) and 7 (*p* < 0.01; Figure 6F) after ischemic injury than in the I/R + sh‐H19 group.

Furthermore, western blot analysis demonstrated a significant upregulation of VEGFR2 protein expression in the I/R + sh‐H19 group than in the I/R + sh‐NC group (*p* < 0.05; Figure 6G,H). Conversely, VEGFR2 expression was reduced in the I/R + sh‐H19 + sh‐IMP2 group relative to the I/R + sh‐H19 group (*p* < 0.05; Figure 6G,H), indicating that inhibiting IMP2 reversed the protective effects of H19 knockdown on angiogenesis post I/R injury.

Similarly, the concentration of claudin5 was significantly higher in the I/R + sh‐H19 group than in the I/R + sh‐NC group (*p* < 0.01; Figure 6I,J). In contrast, claudin5 expression was decreased in the I/R + sh‐H19 + sh‐IMP2 group relative to that in the I/R + sh‐H19 group (*p* < 0.05; Figure 6I,J), suggesting that IMP2 inhibition reversed the protective effects of H19 knockdown on BBB repair following I/R injury.

## 4 DISCUSSION

This study explored the cerebroprotective effects of H19 knockdown in ischemic stroke and its underlying mechanisms. A previous study demonstrated a significant positive correlation between circulating H19 levels and the National Institute of Health Stroke Scale (NHISS) scores of patients, observed at 7, 30, and 90 days post‐ischemic stroke.^10^ This study revealed that increased H19 expression exacerbated cerebral tissue damage and hindered neurological recovery after cerebral I/R injury, which is consistent with previous clinical findings. Additionally, previous research by our team linked increased plasma H19 concentrations to a higher risk of aspirin resistance and recurrent ischemic stroke.^11^ These results highlighted the potential of H19 inhibitors as cerebroprotective agents for IS. Cerebroprotection includes neuroprotection and vasculoprotection among others.^18^ Consequently, we explored the role of H19 knockdown in neuroprotection and vasculoprotection following IS and the underlying mechanisms. The results of our experiments revealed that H19 silencing alleviated cerebral tissue damage and promoted the recovery of neural function following cerebral I/R injury, suggesting that H19 knockdown plays a vital role in neuroprotection. Despite the STAIR guidelines recommending long‐term investigations (2–3 weeks), this study did not perform long‐term neurological function scoring for mice with cerebral ischemia.^19^, ^20^ There are two reasons for this. First, the previous research by our team showed that H19 knockdown is also beneficial for long‐term neurological recovery.^10^ Second, the purpose of this study was not only to investigate the cerebroprotective effect of H19 knockdown after IS. It was also to investigate the downstream mechanisms, where changes in downstream genes and proteins occur rapidly. After comprehensive consideration, we chose 7 days after MCAO as the time point for this study. Furthermore, lncRNA H19 has been identified as a key player in various physiological and pathological processes linked to stroke, including inflammation, apoptosis, autophagy, and neurogenesis.^4^, ^8^, ^10^, ^21^ To the best of our knowledge, the involvement of H19 in angiogenesis and its associated mechanisms have not been documented.

In IS, sudden obstruction of cerebral blood flow leads to the deprivation of essential nutrients and oxygen in the brain, resulting in cell damage or death in the brain.^22^ Accumulating evidence has revealed that the restoration of cerebral blood flow is closely linked to a good prognosis after stroke.^23^ Hence, the timely restoration of cerebral blood flow is currently the most effective strategy for improving the outcomes of patients with acute IS. However, the effects of H19 silencing on cerebral blood flow following cerebral ischemia remain to be elucidated. The results of this study revealed that H19 silencing improved cerebral blood flow on day 1 after I/R. A previous study indicated that cerebral hypoperfusion and ischemia can lead to compensatory vasodilation of cerebral blood vessels and cause a compensatory increase in cerebral blood flow.^24^ Our study revealed that H19 knockdown enhanced the compensatory capacity for cerebral autoregulation.

We explored the vasculoprotective effects of H19 knockdown following IS. The BBB is composed of BMECs, pericytes, and the end‐feet of astrocytes and provides a physical and biochemical barrier between blood circulation and the central nervous system (CNS).^25^ Tight junction proteins, particularly claudin5, are pivotal in maintaining BBB integrity by influencing size selectivity.^26^ Research indicates that BBB breakdown leads to increased expression of claudin5, the predominant tight junction protein in brain endothelial cells.^27^ Moreover, an increasing number of recent studies have examined the significance of ZO‐1. ZO‐1 is essential for the optimal formation of intercellular junction complexes between neighboring endothelial cells that govern BBB integrity.^28^ It has been shown that functional and structural changes in the ZO‐1 protein can give rise to tight junction dissociation and increase intercellular space, leading to increased vascular permeability.^29^ Hence, improving BBB integrity by inhibiting the degradation of tight junction proteins may be promising for mitigating brain injury in ischemic stroke. Several lncRNAs are significantly associated with BBB permeability.^30^ A recent study demonstrated a positive correlation between the plasma lncRNA H19 and tight junction protein concentrations in patients with IS.^31^ We further verified the effect of H19 knockdown on BBB protection in animal experiments. Based on our findings, silencing of H19 led to elevated expression of tight junction proteins like claudin5 and ZO‐1 in the MCAO mouse model, suggesting that H19 mitigated BBB disruption following cerebral ischemia/reperfusion.

Cerebral vascular endothelial cells connected through junction proteins partly constitute the BBB.^32^ Furthermore, endothelial progenitor cells (EPCs), crucial precursors of endothelial cells, can migrate toward areas of injury and ischemia and play a significant role in vessel maintenance and development during angiogenesis.^33^ Angiogenesis, the process of creating new blood vessels from an existing vascular network, is vital for wound healing and combating tissue ischemia.^34^ Angiogenesis in peri‐infarct regions of the brain with IS promotes neurological recovery and is associated with improved post‐stroke survival outcomes.^12^ Endothelial cells secrete multiple vascular endothelial growth factors (VEGFs). The VEGFs, in turn, stimulate angiogenesis by inducing the movement and division of endothelial cells, which is mediated by their VEGF receptors, notably VEGFR2.^35^ Hence, controlling VEGFR2 regulation may be a crucial mechanism governing angiogenesis, given its significant involvement in this process. Previous studies have indicated that VEGFR2, VE‐cadherin, and CD31 act as mechanosensors in angiogenesis and vascular restructuring influenced by shear stress.^36^ Recent reports have highlighted the critical role of lncRNAs in the regulation of angiogenesis during and after IS.^37^, ^38^ However, the effect of H19 on angiogenesis after IS remains unclear. Our investigation revealed that downregulation of H19 notably increased the concentrations of VEGFR2 and CD31 proteins following cerebral I/R injury. Furthermore, we observed an increase in the number of proliferating endothelial cells after cerebral I/R injury following H19 knockdown. These findings confirm that the suppression of H19 enhances angiogenesis after cerebral I/R injury.

We investigated the mechanisms underlying the effects of H19 on angiogenesis after cerebral I/R injury. Using the online prediction tool ENCORI (https://rnasysu.com/encori/), we identified IMP2 as a potential target gene of lncRNA H19. Insulin‐like growth factor 2 mRNA‐binding protein 2 (IMP2), also referred to as IGF2BP2, belongs to a conserved family of RNA‐binding proteins that participate in various cellular biological processes, including cell survival, neural stem/progenitor cell proliferation, and neuronal differentiation.^39^, ^40^, ^41^ One study investigated the role of trimethylamine N‐oxide in ischemic stroke and found it could promote the activation of the NLRP3 inflammasome in microglia through the FTO/IGF2BP2 pathway, thereby aggravating neurological injury in ischemic stroke.^42^ This study suggests that IMP2 is expressed in microglia and may be involved in the inflammatory response during ischemic stroke. Another study demonstrated that propofol treatment inhibited the proliferation, migration, and neuronal differentiation of neural stem cells through miR‐141‐3p/IGF2BP2 signaling.^43^ Moreover, IMP2 is expressed in neural stem/progenitor cells after hypoxic–ischemic brain injury and is highly enriched in the axon tracts.^44^, ^45^ Therefore, IMP2 is likely to be expressed in various cell types, including microglia, neural stem cells, and neurons, during ischemic stroke. These cells play crucial roles in response to ischemic injury and contribute to the pathophysiology of stroke. In this study, western blotting and immunofluorescence staining demonstrated that H19 knockdown increased IMP2 expression in mice with cerebral I/R. To further explore the association between H19 and IMP2, we conducted a rescue experiment. Our findings revealed that silencing IMP2 effectively reversed the protective effects of H19 suppression on the recovery of neurological function, angiogenesis, and BBB repair. The probable mechanisms by which lncRNA H19 regulates the expression of IMP2 include acting as a competing endogenous RNA or transcriptional and post‐transcriptional modulation of gene expression. Here are some potential mechanisms. Regarding the competing endogenous RNA (ceRNA) mechanism, lncRNA H19 can act as a molecular sponge for miRNAs that would bind to the 3′ untranslated region (UTR) of the IMP2 mRNA. This relieves the inhibition of IMP2 translation. Regarding transcriptional regulation, lncRNA H19 may interact with transcription factors or chromatin modifiers to alter the chromatin state at the IMP2 locus, either by promoting or inhibiting its transcription. Regarding post‐transcriptional regulation, lncRNA H19 can influence the stability or translation efficiency of IMP2 mRNA by binding to the mRNA and affecting its processing or degradation. Regarding exosome‐mediated transfer, lncRNA H19 may be packaged into exosomes and transferred to other cells, where it can influence the expression of IMP2 in the recipient cells. Regarding the interaction with proteins, lncRNA H19 may bind proteins involved in the regulation of IMP2, either directly affecting the function of these proteins or serving as a scaffold to bring together multiple regulatory factors.

However, this study had several limitations. First, the study had a modest sample size. Second, the MCAO model is a reliable and consistent tool for studying ischemic stroke, but it does not encapsulate the full complexity of its development and progression.

## 5 CONCLUSION

In summary, the suppression of lncRNA H19 bolstered BBB integrity and stimulated angiogenesis in this study by regulating IMP2 after cerebral I/R injury. Targeting lncRNA H19 is a promising therapeutic strategy for treating IS.

## AUTHOR CONTRIBUTIONS

Yumin Luo, Ping Liu, Liyuan Zhong, and Junfen Fan contributed to the conception and design of the study. Liyuan Zhong, Junfen Fan, Feng Yan, Zhenhong Yang, Yue Hu, and Lingzhi Li contributed to the acquisition and analysis of data. Liyuan Zhong and Junfen Fan contributed to drafting the text and preparing the figures. All authors contributed to the interpretation of the date and drafting the manuscript. All authors reviewed the drafts and approved the final version of the manuscript.

## CONFLICT OF INTEREST STATEMENT

The authors declared no potential conflicts of interest with respect to the research, authorship, and/or publication of this article.

## Data Availability

The data that support the findings of this study are available from the corresponding author upon reasonable request.
